# Maximum-Likelihood Estimator of Clock Offset between Nanomachines in Bionanosensor Networks

**DOI:** 10.3390/s151229830

**Published:** 2015-12-07

**Authors:** Lin Lin, Chengfeng Yang, Maode Ma

**Affiliations:** 1 School of Mechatronic Engineering and Automation, Shanghai University, Shanghai 200072, China; ycfmaple@163.com; 2 School of Electrical and Electronic Engineering, Nanyang Technological University, Singapore 639798; emdma@ntu.edu.sg

**Keywords:** molecular communication, bionanosensor networks, diffusion, clock synchronization

## Abstract

Recent advances in nanotechnology, electronic technology and biology have enabled the development of bio-inspired nanoscale sensors. The cooperation among the bionanosensors in a network is envisioned to perform complex tasks. Clock synchronization is essential to establish diffusion-based distributed cooperation in the bionanosensor networks. This paper proposes a maximum-likelihood estimator of the clock offset for the clock synchronization among molecular bionanosensors. The unique properties of diffusion-based molecular communication are described. Based on the inverse Gaussian distribution of the molecular propagation delay, a two-way message exchange mechanism for clock synchronization is proposed. The maximum-likelihood estimator of the clock offset is derived. The convergence and the bias of the estimator are analyzed. The simulation results show that the proposed estimator is effective for the offset compensation required for clock synchronization. This work paves the way for the cooperation of nanomachines in diffusion-based bionanosensor networks.

## 1. Introduction

Recent advances in the fields of nanotechnology, electronic technology and biology have enabled the development of bionano-devices manufactured in a scale ranging from one to a hundred nanometers [1]. At this scale, a nano-device, also called a bionanosensor or nanomachine, is considered to be the most basic functional device, able to perform tasks at the nano-level, such as computing, data storage, sensing or actuation. Like ordinary sensors and wireless sensor networks, nanomachines can also be interconnected, forming a bionanosensor network [2], to execute more complex tasks and to expand their range of operations [3]. Bionanosensor networks have a broad range of potential applications. For example, in the area of biomedical engineering, bionanosensor networks can be applied in intra-body health monitoring, drug delivery systems, *etc.* In industrial and consumer goods applications, bionano-sensor networks can be used for the development of intelligent functionalized materials, new manufacturing processes, distributed quality control procedures, *etc.* [4].

**Figure 1:**
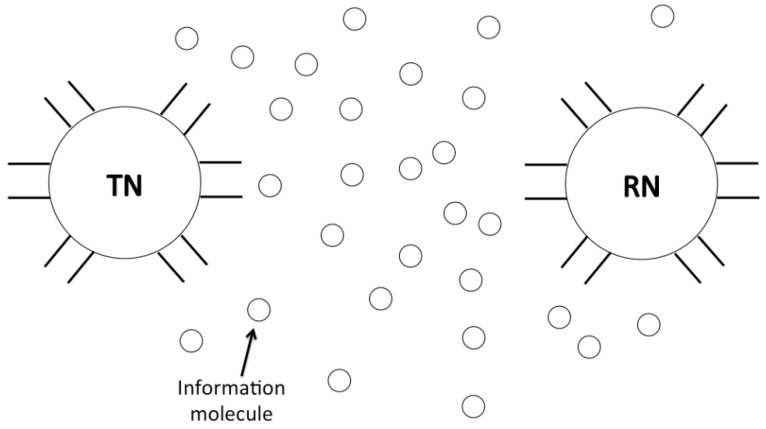
A typical molecular communication system in a bionnanosensor network.

Molecular communication, a kind of bio-inspired communication, is a very popular and promising information exchange technique in the recent nano-communication and bionanosensor network studies [5,6,7,8]. A typical molecular communication system is shown in Figure 1. A transmitter nanomachine sends messages by releasing the information molecules. The information molecules propagate directionally or diffuse randomly in the environment, and reach a receiver nanomachine. The receiver nanomachine then decodes the information molecules into the original message. Since the molecular communication paradigm is different from the conventional radio communication at the physical layer such as channel and signal propagation, the corresponding communication techniques and networking protocols need to be reconsidered and redesigned.

Clock synchronization is a very important issue for all kinds of communication networks, including nano-communication and nanosensor networks. It is the foundation of the distributed collaboration among a set of cooperative nanomachines. For example, in an immune defense bionnanosensor network, the nanomachines are envisioned to release antibody molecules at the same time to effectively kill the pathogen, or in a nanosensor network-based monitoring scenario, clock synchronization is the basis for the interpretation of the sensed data from different nanomachines. However, the slowness and randomness of the propagating molecules represent a big challenge to clock synchronization in bionanosensor networks. The relevant studies of the clock synchronization issues in the wireless sensor networks field can be the good references for the study of clock synchronization in bionanosensor networks [9,10], while those solutions cannot be directly applied to solving the clock synchronization problems in the bionnanosensor networks. The solutions in [11,12] proposed an averaging algorithm for the clock offset compensation for the synchronization in the wireless sensor networks. However, the design ignores the propagation delay of the information exchange between the sensor nodes. Since in bionnanosensor networks, the information molecules propagate much more slowly than electromagnetic waves, the propagation delay is much larger, which cannot be ignored in any algorithm design for clock synchronization. The timing-sync protocol for sensor networks (TPSN) [13] and the recursive time synchronization protocol (RTSP) [14] for wireless sensor networks considered the propagation delay in the design, however, they assume that the propagation delays are bi-directionally symmetrical, while in molecular communications, the molecules move with strong randomness, so the assumption of symmetrical propagation delays is not appropriate for molecular communications. References [15,16] modeled the random propagation delay following a Gaussian distribution. References [16,17,18,19,20] modeled the random delay as an exponential delay and a bivariate exponential delay, but in molecular communications, the propagation delay is normally assumed to follow an inverse Gaussian distribution [21]. In [22], an approach for clock offset estimation has been proposed which is robust to the distribution of the network delays, however, it has high computational complexity and no closed-form expression of the estimator can be obtained.

The oscillation and synchronization of bionanosensor networks have been investigated in recent years. In [23,24], the authors used a bacterial quorum sensing mechanism to synchronize nano-machines. Molecules, called inducers, can be released by one nanomachine, and trigger another nanomachine to release the same self-induced molecules. When the concentration of the inducer particles in the environment reaches a certain threshold at a moment in time, the entire nanonetwork can achieve clock synchronization. In [25,26], clock synchronization was realized by inhibitory molecules. These molecules are released by one transmitter, and inhibit the release of the similar molecules from another transceiver in the nanonetwork. When the concentration of molecules falls below a certain threshold, those molecules can be released again. The pattern of the release pulses of the inhibitory molecules provides the synchronization. However, the abovementioned works try to synchronize the oscillation period rather than align the time. It is crucial and indispensable for the nanomachines to synchronize the time in various nanonetwork applications. In [27], the authors proposed a blind algorithm for such synchronization using non-decision directed maximum likelihood. The clock sequence is calculated by the receiver based on the analysis of the molecular channel delay. However, the authors designed receiver sampling sequences without any further discussion on how to synchronize the time between the transmitter and the receiver.

In this paper, based on the statistical delay model of the molecular diffusion, a two-way message exchange model for clock synchronization is proposed. A maximum likelihood estimator of the clock offset between two nanomachines is designed. We assume that the nanomachines have their own time and the aim of this work is to estimate the time difference between nanomachines. This is the major difference of this paper from the other literature [6,7,8,9,10]. To the best of our knowledge, this is the first work focusing on estimating the clock offsets for diffusion-based nano-communication. The simulation results validate the effectiveness of the proposed algorithm. This work is an important step and the foundation for the distributed cooperation of nanomachines.

The rest of the paper is organized as follows: Section 2 introduces the system model. The maximum likelihood estimator and the analytical analysis are given in Section 3. The Cramer Rao Lower Bound is derived in Section 4. Section 5 presents the simulation results and discussions. Finally, Section 6 concludes the paper.

## 2. System Model

In bionanosensor networks, it is assumed that every individual nanomachine has its own clock. The clock in the nanomachine is analogous to the clock in electronic devices [28]. A clock in an electronic device is considered as a measurement device consisting of an oscillator and memory. Accordingly, the nanomachine components are designed similarly. The memory and processing functionalities in nanomachines are mentioned and designed in [4,29,30]. For example, the nanomachine can use the increase or decrease Ca^2+^ levels, high or low levels of the pH value, or some three dimensional structure to implement the memory function. There are several ways to implement the oscillator. A circadian oscillator is proposed in [31]. In references [32,33] translation and transcription in gene expression is used to generate the oscillation. A molecular phase locked loop (PLL) is proposed in [28] which can be used for the oscillator in a nanomachine. The clock reading of node i at time t, $C_{i}\left(t\right)$, is defined as in Equation (1), where $\beta_{i}$ is the clock offset. In the ideal situation, $\beta_{i}$ is equal to 0, and $C_{i}\left(t\right)$ is equal to the real time *t*:

(1) $$C_{i}\left(t\right)=t+\beta_{i}$$

In order to achieve a distributed cooperation among nanomachines, clock synchronization is required. The objective of clock synchronization is to estimate and compensate the clock offset. For this purpose, one nanomachine can send its clock readings to another nanomachine. Since the message exchange in the nanonetwork is through molecular communication, the information molecules need a quite long period of time to propagate from the transmitter to the receiver. Therefore the propagation delay estimation is necessary.

**Figure 2:**
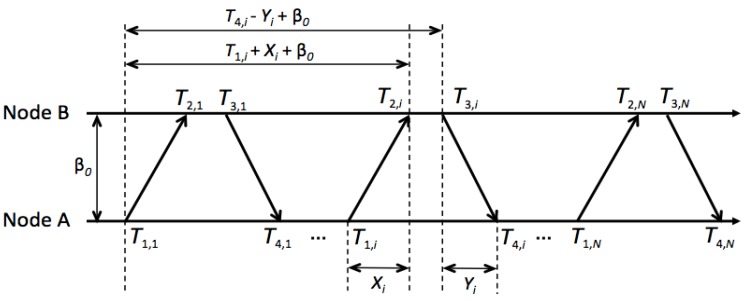
Two-way message exchange for diffusion-based clock synchronization between two nanomachines, node A and node B.

In a molecular communication scenario, we consider a clock synchronization process between two nanomachines, node A and node B. Assume that node A and node B have independent times. The clock offset between them is supposed to be $\beta_{0}$, where $\beta_{0}=\beta_{B}-\beta_{A}$. Node A is synchronized with node B by a two-way timing message exchange mechanism shown in Figure 2. Take the ith round of message exchange as an example. Node A sends a clock synchronization request message. At the same time it records the timing on its own clock as $T_{1,i}$. Node B records its time $T_{2,i}$ at the reception of the message, and replies to node A at $T_{3,i}$. The reply message contains the time stamps $T_{2,i}$ and $T_{3,i}$. Then, node A records the reception time of node B’s reply as $T_{4,i}$. $T_{1,i}$ and $T_{4,i}$ are the time stamps recorded by the clock of node A, while $T_{2,i}$ and $T_{3,i}$ are those recorded by node B. After n rounds of message exchange, node A obtains a set of time stamps ${\left\{T_{1,\;i},\;T_{2,\;i},T_{3,\;i},T_{4,\;i}\right\}}_{i=1}^{N}$. Node A then tries to use these time stamps to estimate the propagation delay of the information molecules. The above procedure can be modeled as:

(2) $$T_{2,\;i}=T_{1,\;i}+\beta_{0}+X_{i}$$

(3) $$T_{3,\;i}=T_{4,\;i}+\beta_{0}-Y_{i}$$

where $\beta_{0}$ denotes the clock offset of node B with respect to node A. $X_{i}$ and $Y_{i}$ are the random variables of the propagation delays from node A to node B and from node B to node A, respectively.

Molecular diffusion is characterized by Brownian motion. It is assumed in this paper that the information is modulated based on the type of the molecules. Each molecule can carry multiple information bits [34] so that the time stamp message can be encoded into an individual molecule. Normally, a Gaussian distribution is used to estimate the distance of the Brownian motion in a fixed period. The inverse Gaussian distribution is used to describe the distribution of the duration a Brownian motion takes to reach a fixed positive distance [35]. The probability density function of an inverse Gaussian distribution random variable can be expressed as:

(4) $$f\left(t;\mu ;\lambda \right)={\left(\frac{\lambda }{2\pi t^{3}}\right)}^{\frac{1}{2}}exp\left[-\frac{\lambda {\left(t-\mu \right)}^{2}}{2\mu^{2}t}\right]$$

where *t* is the random propagation delay, known as the first arrival time [21]. μ is the mean of *t*. λ is the shape parameter. According to [36], $\mu =\frac{d}{v}$ and $\lambda =\frac{d^{2}}{2D}$, where d represents the distance between the nanomachines, *v* is the medium velocity, and *D* represents the diffusion coefficient. $X_{i}$ and $Y_{i}$ are modeled as independent and identical distributed (i.i.d.) inverse Gaussian random variables with a common shape parameter λ. The goal is to estimate the clock offset $\beta_{0}$ based on the observation of a set of time stamps.

## 3. Maximum-Likelihood Estimator

In this section, we propose an approach using maximum- likelihood estimation (MLE) to estimate the clock offset $\beta_{0}$. To derive the MLE, we rewrite Equations (2) and (3) as Equations (5) and (6):

(5) $$X_{i}=T_{2,\;i}-\;T_{1,\;i}\;-\;\beta_{0}$$

(6) $$Y_{i}=-T_{3,\;i}+T_{4,\;i}+\beta_{0}$$

Since ${\left\{X_{i},\;Y_{i}\right\}}_{i=1}^{N}$ are i.i.d. inverse Gaussian random variables, denoting their probability density function as ${\left\{p\left(X_{i}\right),p\left(Y_{i}\right)\right\}}_{i=1}^{N}$, the likelihood function of ${\left\{T_{1,\;i},\;T_{2,\;i},T_{3,\;i},T_{4,\;i}\right\}}_{i=1}^{N}$ is given by $\prod_{i=1}^{N}p\left(x_{i}\right)p\left(y_{i}\right)$. $x_{i}$ and $y_{i}$ are the outcomes of $X_{i}$ and $Y_{i}$. Equations (5) and (6) into the likelihood function, we have the expression:

(7) $$\begin{array}{l} f\left({\left\{T_{1,\;\;i},\;T_{2,\;\;i},T_{3,\;\;i},T_{4,\;i}\right\}}_{i=1}^{N};\beta_{0},\;\mu ,\lambda \right)=\overset{N}{\underset{i=1}{\prod }}{\left(\frac{\lambda }{2\pi {x_{i}}^{3}}\right)}^{\frac{1}{2}}e^{\frac{-\lambda {\left(x_{i}-\mu \right)}^{2}}{2\mu^{2}x_{i}}}{\left(\frac{\lambda }{2{{\pi \text{y}}_{\text{i}}}^{3}}\right)}^{\frac{1}{2}}e^{\frac{-\lambda {\left(y_{i}-\mu \right)}^{2}}{2\mu^{2}y_{i}}}\\ =\prod_{i=1}^{N}({\left(\frac{\lambda }{2\pi {\left(T_{2,\;\;i}-\;T_{1,\;\;i}\;–\;\beta_{0}\right)}^{3}}\right)}^{\frac{1}{2}}e^{\frac{-\lambda {\left(T_{2,\;\;i}-\;T_{1,\;\;i}\;-\;\beta_{0}-\mu \right)}^{2}}{2\mu^{2}\left(T_{2,\;\;i}-\;T_{1,\;\;i}\;-\;\beta_{0}\right)}}\\ \times {\left(\frac{\lambda }{2\pi {\left(-T_{3,\;\;i}+T_{4,\;\;i}+\beta_{0}\right)}^{3}}\right)}^{\frac{1}{2}}e^{\frac{-\lambda {\left(-T_{3,\;\;i}+T_{4,\;\;i}+\beta_{0}-\mu \right)}^{2}}{2\mu^{2}\left(-T_{3,\;\;i}+T_{4,\;\;i}+\beta_{0}\right)}}) \end{array}$$

In the system model, μ and λ are unknown by the nanomachines. They are considered as the nuisance parameters. To estimate $\beta_{0}$, μ and λ should be replaced by the expression of ${\left\{T_{1,i},T_{2,i},T_{3,i},T_{4,i}\right\}}_{i=1}^{N}$ and $\beta_{0}$. A new random variable, $X_{i}+Y_{i}$, is introduced. By this way $\beta_{0}$ can be eliminated. Since $X_{i}$ and $Y_{i}$ are i.i.d. inverse Gaussian random variables, $X_{i}+Y_{i}=T_{4,i}-T_{3,i}+T_{2,i}-T_{1,i}$ also follows the inverse Gaussian distribution, with a mean of 2*μ* and a shaping parameter of 4*λ* [35]. The likelihood function can be expressed as:

(8) $$f\left({\left\{T_{1,\;i},\;T_{1,\;i},T_{3,\;i},T_{4,\;i}\right\}}_{i=1}^{N};\mu ,\lambda \right)=\overset{N}{\underset{i=1}{\prod }}{\left(\frac{4\lambda }{2\pi {\left(T_{4,i}-T_{3,i}+T_{2,i}-T_{1,i}\right)}^{3}}\right)}^{\frac{1}{2}}e^{\frac{-4\lambda {\left(T_{4,i}-T_{3,i}+T_{2,i}-T_{1,i}-2\mu \right)}^{2}}{2{\left(2\mu \right)}^{2}\left({\text{T}}_{4,i}-{\text{T}}_{3,i}+{\text{T}}_{2,i}-{\text{T}}_{1,i}\right)}}$$

The MLE of μ and λ can be obtained by differentiating the logarithm of Equation (8) with respect to μ and λ and setting the results to zero:

$$\{\begin{array}{c} \frac{\partial ln\prod_{i=1}^{N}p\left(x_{i}+y_{i}\right)}{\partial \mu }=0\\ \frac{\partial ln\prod_{i=1}^{N}p\left(x_{i}+y_{i}\right)}{\partial \lambda }=0 \end{array}$$

We get:

$$\{\begin{array}{c} \hat{\mu }=\frac{\sum_{i=1}^{N}\left(T_{4,i}-T_{3,i}+T_{2,i}-T_{1,i}\right)}{2N}\\ \hat{\lambda }=\frac{N}{4\sum_{i=1}^{N}\left(\frac{1}{T_{4,i}-T_{3,i}+T_{2,i}-T_{1,i}}-\frac{N}{\sum_{i=1}^{N}{\left(T_{4,i}-T_{3,i}+T_{2,i}-T_{1,i}\right)}_{i}}\right)} \end{array}$$

We put $\hat{\mu }$ and $\hat{\lambda }$ into Equation (7). The logarithm of the profile likelihood function is expressed as:

(9) $$\begin{array}{l} ln\left(f\left({\left\{T_{1,\;i},\;T_{1,\;i},T_{3,\;i},T_{4,\;i}\right\}}_{i=1}^{N};\beta_{0}\right)\right)\\ =ln\left(\overset{N}{\underset{i=1}{\prod }}p\left(x_{i}\right)p\left(y_{i}\right)\right)\\ =ln\left(\overset{N}{\underset{i=1}{\prod }}{\left(\frac{\lambda }{2\pi {x_{i}}^{3}}\right)}^{\frac{1}{2}}e^{\frac{-\lambda {\left(x_{i}-\mu \right)}^{2}}{2\mu^{2}x_{i}}}{\left(\frac{\lambda }{2\pi {y_{i}}^{3}}\right)}^{\frac{1}{2}}e^{\frac{-\lambda {\left(y_{i}-\mu \right)}^{2}}{2\mu^{2}y_{i}}}\right)\\ =\overset{N}{\underset{i=1}{\sum }}(\frac{1}{2}ln\frac{\lambda }{2\pi {x_{i}}^{3}}+\frac{-\lambda {\left(x_{i}-\mu \right)}^{2}}{2\mu^{2}x_{i}}+\frac{1}{2}ln\frac{\lambda }{2\pi {y_{i}}^{3}}+\frac{-\lambda {\left(y_{i}-\mu \right)}^{2}}{2\mu^{2}y_{i}})\\ =-\frac{3}{2}\overset{N}{\underset{i=1}{\sum }}ln\left(\left(T_{2,\;i}-\;T_{1,\;i}\;–\;\beta_{0}\right)\times \left(-T_{3,\;i}+T_{4,\;i}+\beta_{0}\right)\right)+Nln\frac{\lambda }{2\pi }-N \end{array}$$

The MLE of $\beta_{0}$ can be obtained by differentiating Equation (9) with respect to $\beta_{0}$ and setting the results to zero:

$$\frac{\partial ln\left(\prod_{i=1}^{N}p\left(x_{i}\right)p\left(y_{i}\right)\right)}{\partial \beta_{0}}=0$$

We thus have:

(10) $${\hat{\beta }}_{0}=\frac{\sum_{i=1}^{N}\left(T_{3,i}+T_{2,i}-T_{4,i}-T_{1,i}\right)}{2N}$$

So far, we managed to use ${\left\{T_{1,i},T_{2,i},T_{3,i},T_{4,i}\right\}}_{i=1}^{N}$ to estimate the clock offset $\beta_{0}$ between the two nanomachines. The proof of the convergence of the estimated offset is shown in Equation (11). The estimated offset converges in probability to the true offset value between the transmitter and the receiver:

(11) $$\begin{array}{l} \underset{n\to \infty }{\lim}\left({\hat{\beta }}_{0}-\beta_{0}\right)\\ =\underset{n\to \infty }{\lim}\left(\frac{\sum_{i=1}^{N}\left(T_{3,i}+T_{2,i}-T_{4,i}-T_{1,i}\right)}{2N}-\beta_{0}\right)\\ =\underset{n\to \infty }{\lim}\left(\frac{\sum_{i=1}^{N}\left(2\times \beta_{0}+X_{i}-Y_{i}\right)}{2N}-\beta_{0}\right)\\ =\frac{1}{2}\underset{n\to \infty }{\lim}\left(\frac{1}{N}\overset{N}{\underset{i=1}{\sum }}X_{i}-\;\frac{1}{N}\overset{N}{\underset{i=1}{\sum }}Y_{i}\right)\\ =\frac{1}{2}\left(\mu -\mu \right)=0 \end{array}$$

The estimator is unbiased as proved below. Equations (5) and (6) are used in the derivation:

(12) $$E\left({\hat{\beta }}_{0}\right)=E\left(\frac{\sum_{i=1}^{N}\left(T_{3,i}+T_{2,i}-T_{4,i}-T_{1,i}\right)}{2N}\right)\;=E\left(\frac{\sum_{i=1}^{N}\left(X_{i}+\beta_{0}+\beta_{0}-Y_{i}\right)}{2N}\right)=\frac{\sum_{i=1}^{N}E\left(X_{i}+\beta_{0}+\beta_{0}-Y_{i}\right)}{2N}=\frac{2N\beta_{0}}{2N}=\beta_{0}$$

## 4. Cramer Rao Lower Bound

In this section, the Cramer Rao Lower Bound (CRLB) is derived. The CRLB is a bound of the variance of any unbiased estimator. It is critical to evaluate how much performance loss is incurred by the proposed estimator of the clock offset based on the observations. For the molecular transmission from node A to node B in the ith round of message exchange, the likelihood function of the unknown $\beta_{0}$ is written as:

(13) $$\begin{array}{l} L\left(\beta_{0};\mu \;,\;\lambda \;,\;T_{1,\;i}\;,T_{2,\;i}\right)\\ ={\left(\frac{\lambda }{2\pi {\left(T_{2,\;i}-\;T_{1,\;i}\;–\;\beta_{0}\right)}^{3}}\right)}^{\frac{1}{2}}\times exp\left[-\frac{\lambda {\left(T_{2,\;i}-\;T_{1,\;i}\;-\;\beta_{0}-\mu \right)}^{2}}{2\mu^{2}\left(T_{2,\;i}-\;T_{1,\;i}\;-\;\beta_{0}\right)}\right] \end{array}$$

where *μ* and *λ* are unknown, and $T_{1,\;i}$ and $T_{2,\;i}$ are random variables. The score is defined to indicate how sensitively a likelihood function depends on its parameter. The score for $\beta_{0}$ can be expressed as:

(14) $$V_{\beta_{0}}=\frac{\partial }{\partial \beta_{0}}\log L\left(\beta_{0};\mu \;,\;\lambda \;,\;T_{1,\;i}\;,T_{2,\;i}\right)$$

The variance of the score is known as the Fisher information. It can be expressed as:

(15) $$\begin{array}{l} {\text{FIM}}_{X_{i}}\left(\beta_{0}\right)=-E\left(\frac{\partial^{2}}{\partial {\beta_{0}}^{2}}\log L\left(\beta_{0};\mu \;,\;\lambda \;,\;T_{1,\;i}\;,T_{2,\;i}\right)|\beta_{0}\right)\\ =\;-E\left(\frac{\partial }{\partial \beta_{0}}\left(\frac{3}{2}{X_{i}}^{-1}+\frac{\lambda }{2\mu^{2}}\left(1-\mu^{2}{X_{i}}^{-2}\right)\right)\right)\\ =-E\left(\frac{3}{2}{X_{i}}^{-2}-\lambda {X_{i}}^{-3}\right) \end{array}$$

where $X_{i}=T_{2,\;i}-\;T_{1,\;i}\;-\;\beta_{0}$. The closed-form expression of the expected value is difficult to compute. ${\text{FIM}}_{X_{i}}\left(\beta_{0}\right)$ can be approximated as:

(16) $${\text{FIM}}_{X_{i}}\left(\beta_{0}\right)=-\overset{+\infty }{\underset{x=0}{\sum }}\left(\frac{3}{2}{X_{i}}^{-2}-\lambda {X_{i}}^{-3}\right){\left(\frac{\lambda }{2\pi {X_{i}}^{3}}\right)}^{\frac{1}{2}}e^{\frac{-\lambda {\left(X_{i}-\mu \right)}^{2}}{2\mu^{2}X_{i}}}∆X_{i}$$

For the message transmission from node B to node A, the Fisher information ${\text{FIM}}_{Y_{i}}\left(\beta_{0}\right)$ is calculated. The result is that ${\text{FIM}}_{Y_{i}}\left(\beta_{0}\right)$ is equal to ${\text{FIM}}_{X_{i}}\left(\beta_{0}\right)$. For the total *N* round bi-directional message exchanges, the fisher information is expressed as:

(17) $$\text{FIM}\left(\beta_{0}\right)=N*\left({\text{FIM}}_{X_{i}}\left(\beta_{0}\right)+{\text{FIM}}_{Y_{i}}\left(\beta_{0}\right)\right)\;=-2N\overset{+\infty }{\underset{x=0}{\sum }}\left(\frac{3}{2}{X_{i}}^{-2}-\lambda {X_{i}}^{-3}\right){\left(\frac{\lambda }{2\pi {X_{i}}^{3}}\right)}^{\frac{1}{2}}e^{\frac{-\lambda {\left(X_{i}-\mu \right)}^{2}}{2\mu^{2}X_{i}}}∆X_{i}$$

The CRLB is the inverse of the fisher information. The variance of the estimator will be greater than or equal to the CRLB as:

(18) $$var\left({\hat{\beta }}_{0}\right)\geq \frac{1}{\text{FIM}\left(\beta_{0}\right)}$$

## 5. Simulation Results and Discussions

To evaluate the proposed maximum-likelihood estimator for the clock offset, extensive simulation experiments using MATLAB have been conducted. Suppose that there are two nanomachines, a transmitter nanomachine and a receiver nanomachine. They are separated by a distance of 1–10 μm, which is a typical distance used in [37,38]. The medium velocity is set to 1–5 μm/μs [21,39]. Using the equation $\mu =\frac{d}{v}$ mentioned in Section 2, we choose the average propagation delay of the information molecules from 1 to 5 μs which is within the feasible range. The diffusion coefficient *D* is set to 1–10 μm^2^/μs, which is used in [36,40]. According to the equation $\lambda =\frac{d^{2}}{2D}$ mentioned in Section 2, we choose the shape parameter λ=1, which is within the feasible range. Assume that at each integral time instant, the transmitter nanomachine sends a timing-request command to the receiver nanomachine. The propagation delays of the message transmissions between the transmitter nanomachine and the receiver nanomachine are generated based on the inverse Gaussian distribution with mean *μ* and the shape parameter *λ*. Since the simulation aims to evaluate the performance of the proposed maximum likelihood estimator for the clock offset based on the system model in Equations (2) and (3), we generate the propagation delay directly based on the inverse Gaussian distribution. The initial clock offset in the simulation is set to 100 μs to 300 μs. The simulations are performed 100 times. The mean square error (MSE) of the clock offsets is calculated.

**Figure 3:**
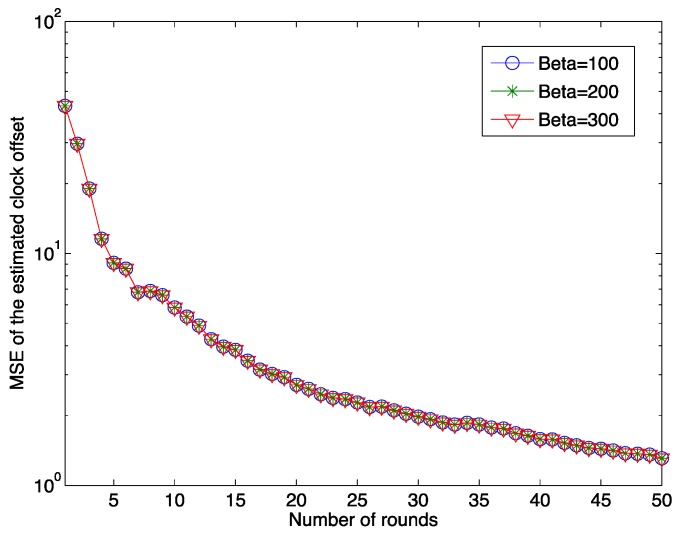
The MSE of the estimated offset as a function of the number of rounds for different initial clock offsets when μ = 5 μs and λ = 1.

Figure 3 shows the relationship between the MSE of the estimated offset and the number of rounds for different pre-defined $\beta_{0}$ values. It is clear that as the number of synchronization rounds increases, the MSE of the estimated clock offset decreases quickly. This result illustrates the effectiveness of our proposed estimation algorithm. It should also be noted that after 50 rounds of the synchronization, the MSE still does not reach zero. In the figure, the MSEs of the estimated clock offset for three different initial clock offset values, 100, 200 and 300 μs, are presented. The result implies that the initial clock offset does not affect the clock synchronization accuracy by the proposed clock synchronization algorithm.

**Figure 4:**
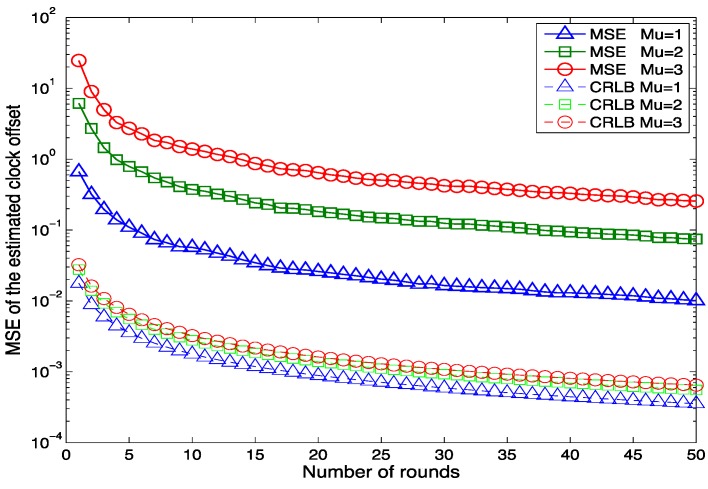
The MSE of the estimated offset as a function of the number of rounds with various delays when $\beta_{0}$ = 100 μs and λ =1.

Figure 4 shows the relationship between the MSE of the estimated clock offset and the number of clock synchronization rounds with different means of the propagation delay μ. Like Figure 3, the MSE of the estimated clock offset decreases as the number of synchronization rounds increases for all the different propagation delays. This also proves the effectiveness of our proposed algorithm. It is obvious that the larger the mean propagation delay is, the larger the MSE of the estimated clock offset is. The reason for this fact is that if the mean of the propagation delay is larger, then there will be a longer period for the information molecules to move randomly in the environment. The delay will be more diverse and decrease the clock synchronization accuracy. Generally speaking, a larger mean propagation delay is due to a longer distance between the nanomachines. Therefore, a densely deployed nanonetwork could achieve a better clock synchronization performance than a loosely deployed nanonetwork in terms of accuracy.

The CRLBs of the estimated clock offset for different average delays are also drawn in this figure. It is clear that the CRLB becomes smaller as the number of message exchange rounds increases. This can be explained by Equation (16). Due to the additivity property of the Fisher information [41], as long as the experiments are independent, every observation contains the information about the unknown parameter. Therefore, as *N* increases, more information about $\beta_{0}$ is obtained. Then the CRLB becomes smaller. From Figure 4, it can also be seen that the MSE of the estimated clock offset is bigger than the CRLB. This implies that the proposed estimator is not the minimum variance unbiased (MVU) estimator. The work can be continued to find the MVU estimator of the clock offset.

**Figure 5:**
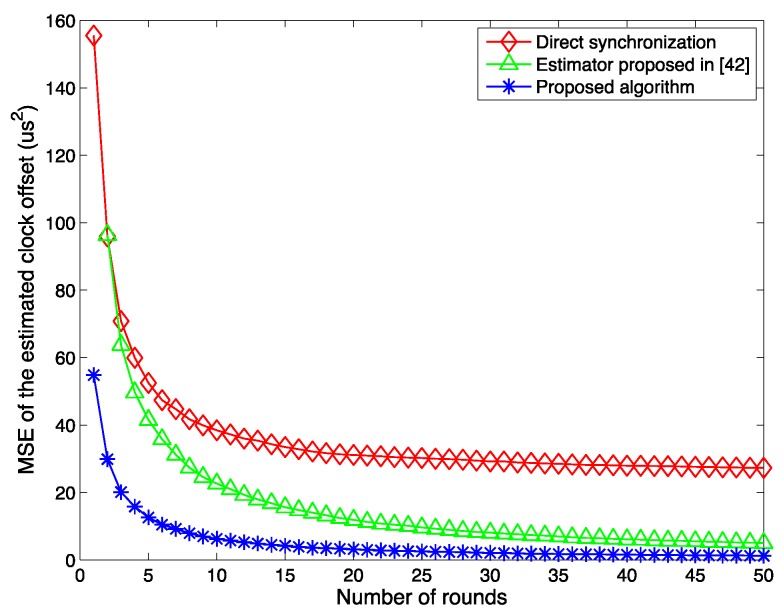
Comparison among the proposed algorithm, direct synchronization, and estimator proposed in [42]; $\beta_{0}$ = 100 μs, μ = 5 μs and λ = 1.

Figure 5 compares the MSEs of the estimated clock offsets by using the proposed MLE algorithm, the direct synchronization, and the estimator proposed in [42]. In the direct synchronization algorithm, the receiver ignores the propagation delay, and considers that the difference between its recorded receiving time stamp and the received transmitting time stamp from the transmitter is the clock offset. The algorithm in [42] is similar to the algorithm proposed in this paper but considers both the clock offset and the clock skew estimation. For fair comparison, all the three algorithms use the same input data in the simulations. From the figure, it can be seen that as the number of rounds increases, the estimated offsets become smaller, which manifests the effectiveness of all three algorithms. The MSEs of the estimated clock offset by the proposed MLE scheme is smaller than that by the other two schemes. This proves that the proposed MLE can achieve a better accuracy performance. The reason that the direct synchronization has the biggest MSE is because the propagation delay is neglected in the calculation. The model is simplified compared to the other two algorithms, while in a real scenario, the molecular communication incurs a propagation delay, which affects the estimation significantly. The reason that the proposed algorithm outperforms the estimator described in [42] is because the estimation in [42] is a joint estimation for the clock offset and the clock skew. The result is obtained when both of the two estimators achieve their maximum likelihood. For the estimation of the clock offset in this paper, the proposed estimator is derived only for the clock offset itself achieving the maximum likelihood. Therefore, the proposed solution gives better performance.

Table 1 summarizes the computational complexity of the three estimators. The numbers of the operations for the clock synchronization by the nanomachine or sensor node are shown in the table. The values are calculated based on the expressions of the different estimators. From the figure it is clear that the direct synchronization method uses fewer operations than the other two estimators. But the performance in terms of the accuracy is the worst, which is obtained in Figure 5. The complexity of our proposed algorithm and the estimator proposed in [42] are both O(N).

**Table 1:** Complexity comparison of different algorithms.

	No. of Operations	Number of +/−	Number of ×	Number of ÷
Estimator				
Direct synchronization		2N	0	1
Estimator proposed in [42]		7N − 5	5N + 3	1
Proposed algorithm		4N	N	1

## 6. Conclusions

In this paper, we have addressed the issue of clock synchronization among nanomachines in bionanosensor networks. A two-way packet-based synchronization method has been proposed. The maximum-likelihood estimator for the clock offset between nanomachines has been derived. The simulation results have demonstrated the effectiveness of the proposed scheme. The convergence and the bias of the estimator have also been verified. The work presented in this paper is a very important step towards achieving distributed cooperation among nanomachines in bionanosensor networks. Future work will focus on solutions to achieve global clock synchronization in nanosensor networks.
